# The roll-your-own cigarette market in Canada: a cross-sectional exploratory study

**DOI:** 10.1186/1617-9625-5-5

**Published:** 2009-03-16

**Authors:** Scott T Leatherdale, Murray Kaiserman, Rashid Ahmed

**Affiliations:** 1 Department of Population Studies and Surveillance, Cancer Care Ontario, Toronto, ON, Canada; 2 Department of Health Studies and Gerontology, University of Waterloo, Waterloo, ON, Canada; 3 Dalla Lana School of Public Health, University of Toronto, Toronto, ON, Canada; 4 Tobacco Control Programme, Health Canada, Ottawa, ON, Canada; 5 Population Health Research Group, University of Waterloo, Waterloo, ON, Canada

## Abstract

**Background:**

Even though the use and prevalence of roll-your-own cigarettes (RYO) has been declining over the past decades, RYO remains important. Given the paucity of research examining RYO use, there is a need to better understand the current and potential future context of RYO use.

**Methods:**

Data from the 2002 Canadian Tobacco Use Monitoring Survey (CTUMS) were used to examine RYO tobacco use among 23,341 Canadians aged 15 and older. Logistic regression models were conducted to examine factors which differentiate smokers who smoke RYO tobacco all of the time, most of the time or sometimes from smokers who do not smoke RYO tobacco.

**Results:**

We found that 17% (n = 925,000) of current smokers in Canada reported smoking RYO. When compared to manufactured cigarette (MC) smokers, RYO users were heavier smokers, more addicted to nicotine, and less likely to consider quitting smoking. Lower income smokers were more likely to smoke RYO tobacco compared to smokers with high income. Conversely, smokers who had completed secondary school or university were less likely to smoke RYO tobacco compared to smokers who had not completed secondary school.

**Conclusion:**

This study demonstrates that RYO tobacco use is not a negligible problem within Canada and provides valuable new insight for developing future tobacco control initiatives for this population of smokers.

## Introduction

Even though the use of roll-your-own cigarettes (RYO) has declined in recent decades [1,2], RYO remains important. For instance, RYO smokers tend to believe that RYO cigarettes are less harmful compared to factory-made (FM) cigarettes [1] despite evidence to suggest that RYO smokers are actually at increased risk for certain cancers [3-5]. RYO smokers also tend to have lower incomes than smokers of FM cigarettes [1], and since fine-cut tobacco used to make RYO cigarettes are taxed at a lower rate than FM cigarettes in Canada, smokers may compensate for price increases by shifting from FM cigarettes to RYO instead of quitting or reducing consumption [2]. Moreover, when you consider that RYO smokers tend to be heavier smokers and less likely to consider quitting smoking compared to FM cigarette smokers [1], it is apparent that this high-risk smoking population should be a priority for tobacco control.

Another reason why RYO use remains important is that there is evidence from Europe that tobacco advertisements are starting to target the young and 'hip' market segments with the 'benefits' of smoking RYO (i.e., it is cheaper and cooler than smoking FM cigarettes) [6,7]. Although these marketing activities are not yet evident in Canada, there is the possibility that popular culture spill-over could encourage young Canadian smokers to experiment more with RYO. Due to the paucity of research examining RYO use, there is a need to better understand the current and potential future context of RYO use in Canada. As such, this paper characterizes the prevalence of RYO smoking in Canada, and identifies characteristics associated with RYO tobacco use among smokers.

## Method

The analyses used data from the 2002 Canadian Tobacco Use Monitoring Survey (CTUMS) [8]. CTUMS is a nationally representative telephone survey of smoking behaviour administered by Statistics Canada to monitor trends in smoking prevalence. Data for the current analysis were drawn from interviews conducted between February and December of 2002. The target population for CTUMS is all persons aged 15 and older (young adults aged 15–24 are over-sampled) living in Canada, excluding residents of Yukon, Nunavut, and the Northwest Territories, and full-time residents of institutions. Data were collected by Statistics Canada between February and December 2002 using computer-assisted interviews by telephone; only direct reports (i.e., not third-party) with selected persons were accepted. Data were collected using informed consent in accordance with Health Canada's ethical guidelines. To allow provincial comparisons of approximately equal reliability, the overall sample size for the survey was divided equally across all 10 Canadian provinces. With this sampling frame, it is possible to estimate the smoking prevalence of Canadians aged 15 and older within about ± 0.9% each year. A total of 23,341 Canadians responded to the survey with an overall response rate of 82%. Survey weights were used to adjust for non-response between provinces and groups, thereby minimizing any bias in the analyses caused by differential response rates across different regions or groups. A full description of the sampling design is available [9,10].

Among current smokers (smoked > 100 cigarettes lifetime and at least once in the past 30 days), daily smokers were those who reported smoking daily and occasional smokers were those who smoked at least once in the past 30 days but not daily. Daily and occasional smokers were asked about quit attempts in the past year, intentions to quit smoking in the next six months, if more expensive cigarettes would make them quit smoking, and to report their cigarette consumption for each of the previous seven days. Time to first cigarette in the morning was used as a proxy measure for nicotine addiction [11]. Sociodemographic information about age, sex, marital status, education and income adequacy (based on household income and household size) were also collected.

CTUMS data were weighted on sex, age, and province, followed by adjustments for non-response and multiple telephone lines within a household. In addition, variance estimates were adjusted using coefficients of variation to take the survey's design effect into account [9,10] In Step 1, descriptive analyses of RYO use and respondent sociodemographic and behavioural characteristics were examined. Chi-square was used to test for significant differences (p < 0.05) across groups. In Step 2, an ordinal logistic regression model was fitted to examine the characteristics which were associated with different levels of RYO tobacco use among current smokers. However, when we tested the assumption of parallel regression using the Chi-square test in our preliminary ordinal model, we identified that the proportionality assumption failed. As such, in order to better understand the characteristics associated with different frequencies of RYO use behavior, we used a more traditional yet robust modeling approach in which three logistic regression models were conducted to examine characteristics which differentiated current smokers who use RYO tobacco (a) all of the time versus never, (b) most of the time versus never, and (c) sometimes versus never. All analyses were conducted using SAS Version 9.1 [12].

## Results

In 2002, 21% of Canadians aged 15 and older were current smokers. Among these 5.5 million smokers, 17% (n = 925,000) reported smoking RYO; 8% (n = 452,000) all of the time, 3% (164,000) most of the time, and 6% (309,000) sometimes. When compared to FM cigarette smokers, RYO users were heavier smokers. The average number of cigarettes per day for those who smoke RYO all of the time was 19.2 (± 9.2) and for those who smoke RYO most of the time was 20.1 (± 8.2) compared to 15.3 (± 6.9) for those who smoke RYO sometimes and 13.8 (± 9.8) for those who never smoke RYO tobacco.

As shown in Table 1, older smokers were more likely to smoke RYO than younger smokers (χ^2 ^= 138.4, *df *= 9, p < .001), as were daily smokers compared to occasional smokers (χ^2 ^= 89.4, *df *= 3, p < .001); 92% of occasional smokers had never smoked RYO compared to only 81% of daily smokers. RYO smoking was not significantly different between males and females (χ^2 ^= 3.4, *df *= 3, p = .336). A smaller percentage of divorced or separated respondents report never smoking RYO (77% and 77% respectively) compared to married or single respondents (83% and 84% respectively), even though married and single respondents represent the largest population of RYO users (n = 187,000 and n = 116,000 respectively). Not only were RYO smokers more addicted to nicotine compared to non-RYO smokers based on their time to smoking after waking up (χ^2 ^= 133.3, *df *= 9, p < .001), but they were also less likely to consider quitting smoking (χ^2 ^= 116.1, *df *= 3, p < .001) or to have made fewer quit attempts (χ^2 ^= 35.1, *df *= 3, p < .001). Nevertheless, beliefs about more expensive cigarettes causing smoking cessation were not significantly different among RYO and non-RYO smokers (χ^2 ^= 4.8, *df *= 3, p = .187). Smokers who had less education tended to smoke RYO more frequently compared to smokers with more education (χ^2 ^= 133.7, *df *= 9, p < .001), as did smokers with lower income adequacy compared to smokers with higher income adequacy (χ^2 ^= 192.9, *df *= 12, p < .001).

**Table 1:** Weighted sample characteristics by roll-your-own (RYO) tobacco use among Canadian smokers, 2002

		**Roll-your-own (RYO) Tobacco Use**				
		**All of the time**	**Most of the time**	**Sometimes**	**Never**	**Chi-Squar**
		**% (n)**	**% (n)**	**% (n)**	**% (n)**	
Smoking status	Daily smoker	95.9 (433,969)	94.2 (154,724)	83.6 (257,901)	79.9 (3,494,082)	χ^2 ^= 89.4,
	Occasional smoker	4.1 (18,408)	5.8 (9,579)	16.4 (50,684)	20.1 (877,067)	*df *= 3, p < .001
Sex	Male	53.2 (240,638)	52.5 (86,208)	58.0 (178,883)	52.6 (2,299,092)	χ^2 ^= 3.4,
	Female	46.8 (211,739)	47.5 (78,095)	42.0 (129,702)	47.4 (2,072,057)	*df *= 3, p = .336
Age (in years)	15–24	9.2 (41,771)	19.5 (32,082)	36.2 (111,788)	20.6 (901,027)	χ^2 ^= 138.4,
	25–34	12.7 (57,406)	20.4 (33,497)	13.1 (40,309)	23.3 (1,016,317)	*df *= 9, p < .00
	35–44	32.7 (147,741)	24.0 (39,458)	17.5 (53,955)	23.2 (1,014,262)	
	45+	45.4 (205,459)	36.1 (59,266)	33.2 (102,533)	32.9 (1,439,543)	
						
Marital status	Common law	14.6 (65,122)	26.5 (43,182)	4.8 (14,216)	11.9 (514,612)	χ^2 ^= 108.8,
	Married	42.0 (187,460)	41.1 (67,132)	32.2 (95,747)	40.7 (1,758,185)	*df *= 15, p < .00
	Widowed	2.4 (10,511)	2.8 (4,588)	5.3 (15,758)	3.7 (159,918)	
	Divorced	10.6 (47,398)	5.7 (9,362)	7.0 (20,735)	6.0 (257,692)	
	Separated	4.5 (19,968)	1.9 (3,109)	2.4 (7,365)	2.3 (101,456)	
	Single	25.9 (115,610)	22.0 (35,859)	48.3 (143,604)	35.4 (1,528,765)	
						
Time to first cigarette after waking up	Within 5 minutes	34.2 (149,146)	27.0 (41,920)	35.3 (95,899)	22.1 (795,446)	χ^2 ^= 133.3,
	6–30 minutes	45.1 (197,095)	30.5 (47,328)	27.3 (74,242)	31.6 (1,135,698)	*df *= 9, p < .00
	31–60 minutes	10.0 (43,581)	22.4 (34,789)	11.6 (31,454)	19.4 (696,273)	
	> 60 minutes	10.7 (46,844)	20.1 (31,294)	25.8 (70,006)	26.9 (967,622)	
						
Intends to quit smoking in the next 6 months	Yes	36.7 (158,390)	63.9 (103,630)	68.2 (202,304)	62.2 (2,606,495)	χ^2 ^= 116.1,
	No	63.3 (273,105)	36.1 (58,606)	31.8 (94,358)	37.8 (1,582,967)	*df *= 3, p < .001
						
More expensive cigarettes would make me quit smoking	Yes	6.5 (23,740)	6.4 (8,656)	9.0 (21,918)	5.7 (195,686)	χ^2 ^= 4.8,
	No	93.5 (340,607)	93.6 (127,172)	91.0 (222,698)	94.3 (3,268,054)	*df *= 3, p = .187
						
Number of quit attempts in last year	None	58.5 (260,665)	49.6 (80,984)	53.6 (163,167)	54.0 (2,238,550)	χ^2 ^= 35.1,
	1 quit attempt	19.3 (86,168)	22.1 (36,185)	14.0 (42,448)	15.8 (657,282)	*df *= 3, p < .001
	2 quit attempts	13.1 (58,597)	9.0 (14,746)	7.6 (23,189)	11.9 (494,079)	
	3+ quit attempts	9.1 (40,366)	19.3 (31,519)	24.8 (75,412)	18.3 (758,947)	
						
Highest level of education attained	Less than secondary	39.4 (175,193)	50.2 (82,557)	37.8 (115,716)	23.9 (1,032,202)	χ^2 ^= 133.7,
	Completed secondary	38.6 (171,849)	35.0 (57,458)	43.5 (133,321)	46.3 (2,003,667)	*df *= 9, p < .001
	Completed college	14.0 (62,568)	12.1 (19,901)	10.2 (31,150)	16.5 (711,383)	
	Completed university	8.0 (35,368)	2.7 (4,387)	8.5 (26,164)	13.3 (575,472)	
						
Income adequacy	Low	36.8 (126,710)	34.2 (41,179)	35.7 (75,644)	20.8 (621,211)	χ^2 ^= 192.9,
	Medium low	45.5 (156,568)	43.4 (52,386)	41.0 (87,006)	32.0 (957,706)	*df *= 12, p < .00
	Medium	13.2 (45,324)	16.2 (19,551)	13.6 (28,909)	21.7 (650,210)	
	Medium high	4.1 (14,194)	5.7 (6,905)	4.2 (8,879)	13.2 (396,370)	
	High	0.4 (1,481)	0.5 (570)	5.5 (11,644)	12.3 (369,533)	

### Factors associated with using RYO tobacco all of the time versus never

As shown in Table 2, smokers aged 35 to 44 (OR 2.57, 95%CI 1.55 to 4.28) or 45 and older (OR 2.47, 95%CI 1.51 to 4.05) were more likely to smoke RYO tobacco all of the time compared to young adults aged 15 to 24. Smokers with middle income (OR 7.35, 95%CI 4.02 to 13.50) or low income (OR 13.07, 95%CI 7.31 to 25.70) were substantially more likely to smoke RYO tobacco all of the time compared to smokers with high income. A smoker was also more likely to smoke RYO all of the time as their frequency of smoking increased (OR 1.03, 95%CI 1.01 to 1.05). Smokers who have their first cigarette within five minutes (OR 2.31, 95%CI 1.40 to 3.81) or six to 30 minutes (OR 2.64, 95%CI 1.67 to 4.18) of waking up were more likely to smoke RYO all of the time compared to smokers who wait more than 60 minutes to smoke their first cigarette. Conversely, smokers who had completed secondary school were less likely to smoke RYO all of the time compared to smokers who had not completed secondary school (OR 0.67, 95%CI 0.49 to 0.91).

**Table 2:** Logistic regression analyses examining factors related to roll-your-own (RYO) tobacco use among Canadian smokers, 2002

		**Adjusted Odds Ratio^§ ^(95% CI)**		
		**Model 1^✠^**	**Model 2^✠^**	**Model 3^✠^**
		*All of the time vs. Never*	*Most of the time vs. Never*	*Sometimes vs. Never*
Sex	Female	1.00	1.00	1.00
	Male	0.84 (0.64,1.11)	0.65 (0.42,1.01)	1.34 (0.93,1.92)
				
Age (in years)	15–24	1.00	1.00	1.00
	25–34	1.48 (0.85,2.58)	1.58 (0.77,3.24)	0.38 (0.21,0.69)**
	35–44	2.57 (1.55,4.28)***	1.01 (0.47,2.15)	0.59 (0.35,0.99)*
	45+	2.47 (1.51,4.05)***	1.61 (0.85,3.07)	0.80 (0.51,1.26)
				
Smoking status	Occasional smoker	1.00	1.00	1.00
	Daily smoker	1.99 (0.64,6.13)	1.22 (0.36,4.21)	0.57 (0.29,1.14)
				
More expensive cigarettes would make me quit smoking	No	1.00	1.00	1.00
	Yes	1.06 (0.62,1.79)	1.50 (0.70,3.18)	1.53 (0.86,2.73)
				
Highest level of education attained	Less than secondary	1.00	1.00	1.00
	Completed secondary	0.67 (0.49,0.91)*	0.45 (0.28,0.73)**	0.53 (0.36,0.78)**
	Completed college	0.69 (0.45,1.07)	0.61 (0.32,1.15)	0.30 (0.15,0.60)***
	Completed university	0.94 (0.56,1.57)	0.13 (0.03,0.67)*	0.44 (0.21,0.93)*
				
Income adequacy	High/Medium high	1.00	1.00	1.00
	Medium/Medium low	7.35 (4.02,13.5)***	3.49 (1.56,7.78)**	2.45 (1.39,4.31)**
	Low	13.07 (7.31,25.7)***	5.22 (2.22,12.3)***	3.18 (1.72,5.88)***
				
Average number of cigarettes per day	Each cigarette	1.03 (1.01,1.05)**	1.06 (1.03,1.09)***	0.98 (0.96,1.01)
						
Time to first cigarette after waking up	More than 60 minutes	1.00	1.00	1.00
	31–60 minutes	0.91 (0.50,1.65)	0.46 (0.22,0.97)*	0.44 (0.22,0.89)*
	6–30 minutes	2.64 (1.67,4.18)***	0.61 (0.34,1.12)	1.38 (0.84,2.25)
	Within 5 minutes	2.31 (1.40,3.81)**	0.61 (0.32,1.16)	2.02 (1.19,3.44)**
				
Number of quit attempts in last year	Each quit attempt	0.93 (0.87,1.04)	1.05 (0.98,1.07)	1.02 (0.99,1.05)

Note: § Odds ratios adjusted for all other variables in the table

Model 1 - 1 = All of the time (n = 309), 0 = Never (n = 1,605); *c *statistic = 0.740

Model 2 - 1 = Most of the time (n = 143), 0 = Never (n = 1,605); *c *statistic = 0.703

Model 3 - 1 = Sometimes (n = 227), 0 = Never (n = 1,605); *c *statistic = 0.660

*p < .05 **p < .01 ***p < .001

### Factors associated with using RYO tobacco most of the time versus never

Smokers with middle income (OR 3.49, 95%CI 1.56 to 7.78) or low income (OR 5.22, 95%CI 2.22 to 12.30) were more likely to smoke RYO most of the time compared to smokers with high income adequacy. Conversely, smokers who had completed secondary school (OR 0.45, 95%CI 0.28 to 0.73) or university (OR 0.13, 95%CI 0.03 to 0.67) were much less likely to smoke RYO most of the time compared to smokers who had not completed secondary school. A smoker was also more likely to smoke RYO most of the time as their frequency of smoking increased (OR 1.06, 95%CI 1.03 to 1.09). Smokers who have their first cigarette within 31 to 60 minutes of waking up were also less likely to smoke RYO most of the time (OR 0.46, 95%CI 0.22 to 0.97) compared to smokers who wait more than 60 minutes to smoke their first cigarette.

### Factors associated with using RYO tobacco sometimes versus never

Smokers aged 25 to 34 (OR 0.38, 95%CI 0.21 to 0.69) or 35 to 44 (OR 0.59, 95%CI 0.35 to 0.99) were less likely to smoke RYO sometimes compared to younger smokers. Similarly, smokers who completed secondary school (OR 0.53, 95%CI 0.36 to 0.78), college (OR 0.30, 95%CI 0.15 to 0.60) or university (OR 0.44, 95%CI 0.21 to 0.93) were also less likely to sometimes smoke RYO compared to smokers who did not completed secondary school. Although smokers who have their first cigarette within 31 to 60 minutes of waking up are less likely to sometimes smoke RYO than smokers who wait more than an hour to have their first cigarette (OR 0.44, 95%CI 0.22 to 0.89), smokers who have their first cigarette within five minutes of waking up are more likely to sometimes smoke RYO (OR 2.02, 95%CI 1.19 to 3.44). Compared to smokers with high incomes, smokers with middle (OR 2.45, 95%CI 1.39 to 4.31) or low incomes (OR 3.18, 95%CI 1.72 to 5.88) were more likely to smoke RYO sometimes.

## Discussion

A very limited amount of research has examined the issue of RYO tobacco use. Regardless, this study demonstrates that RYO tobacco use is not a negligible problem within Canada. In light of this information, and knowing that these 2002 data are the most recent nationally representative data on RYO use available in Canada, we feel that RYO tobacco use needs to become re-integrated into tobacco control surveillance and evaluation activities.

These results indicate that the income adequacy of a smoker had the largest effect on differentiating those who smoke RYO from those who smoke FM cigarettes. For instance, smokers with low and even middle income adequacy were substantially more likely to smoke RYO than higher income smokers. Although previous research had suggested that income difference do not predict exclusive RYO use [1], our findings clearly indicate that in a larger nationally representative sample of smokers, income differences do predict exclusive RYO use. The importance of income suggests that as long as a discrepancy in the excise tax on FM and fine-cut tobacco exists, smokers may compensate for price increases by shifting from factory-made to RYO instead of quitting [2]. This may explain our finding that RYO and FM tobacco use did not vary by beliefs about increased costs of cigarettes leading to cessation. Additional research is required to model how changes in the relative costs of FM and fine-cut tobacco would impact switching from FM to RYO cigarettes or lead to cessation. Such insight will be particularly important if Canadian tobacco manufacturers follow the lead of the UK tobacco industry and position RYO as a cheaper alternative for smokers [6].

Consistent with existing research [1], we found that RYO smokers appear to be more addicted to smoking than MF cigarette smokers based on time to smoking after waking up. We also identified that RYO smokers tend to be heavier smokers than those who smoke FM cigarettes. As such, even though RYO smokers represent a small portion of the entire smoking population, knowing that they are both 'more addicted' and heavier smokers suggests that they may actually be at increased risk for smoking related morbidity and mortality [3-5,13]. Additional research is required to tailor appropriate cessation interventions to this high-risk population.

While older smokers reported more frequent use of RYO tobacco, we also identified that almost 80% of smokers aged 15 to 24 reported having tried RYO, with more than a quarter of young adult smokers reporting frequent RYO use. Since smokers are most apt to switch from FM cigarettes to RYO in younger age groups [1], and our data suggest that smokers within this age group are experimenting with RYO, this is cause for concern. When coupled with increasing prices of FM cigarettes (remembering that youth are the most price sensitive smokers [14]), and evidence that some tobacco manufacturers are starting to target marketing initiatives regarding the 'benefits' of RYO tobacco relative to FM cigarettes to young adult populations [6], this may represent the beginning of a potential future resurgence in RYO use if left unchecked. As such, ongoing surveillance of RYO use and marketing of RYO products, especially among youth populations, is required.

The finding that the use of RYO tobacco did not significantly vary by sex was unexpected. Conventional wisdom has always suggested that more men use RYO than women. More recently, it was identified that although a high proportion of female smokers report mixed use of both RYO and FM cigarettes, the majority of RYO use in developed countries occurs among male smokers [1]. It is possible that this may be a result of the availability of RYO products in Canada that are easier to assemble than the traditional product. Nevertheless, our finding warrants additional research as we need to clearly understand the factors that help to explain why in Canada, rates of RYO are similar between males and females.

### Limitations

This study has several limitations common to survey research. Although the response rate was high and the data were weighted to help account for non-response, the findings are nevertheless subject to sample bias. It should also be noted that the cross-sectional nature of the design does not allow for causal inferences regarding the association between sociodemographic characteristics and RYO tobacco use. Longitudinal data are required.

## Conclusion

RYO tobacco use has come to represent a small and shrinking market in Canada, but it is still responsible for tobacco related morbidity and mortality as more than one in ten Canadian smokers frequently smoked RYO cigarettes in 2002. Not only do RYO smokers tend to be heavier more addicted smokers, but they also tend to be older, and have less income and education than smokers who consume FM cigarettes. Considering that RYO use is also evident among younger populations, and RYO use poses a growing threat to public health internationally, it is clear that the RYO market should not be ignored.

## Competing interests

The authors declare that they have no competing interests.

## Authors' contributions

SL oversaw the analysis, interpretation of results, and writing of the manuscript. MK contributed to interpreting the results and writing the manuscript. RA preformed the analysis and helped with the interpretation of the results.
